# Modulating the Activity of MPFC With tDCS Alters Endowment Effect

**DOI:** 10.3389/fnbeh.2019.00211

**Published:** 2019-09-12

**Authors:** Wenmin Guo, Jinchuan Shi, Xinbo Lu, Hang Ye, Jun Luo

**Affiliations:** ^1^School of Economics, Zhejiang University, Hangzhou, China; ^2^Interdisciplinary Center for Social Sciences, Zhejiang University, Hangzhou, China; ^3^Academy of Financial Research, Zhejiang University, Hangzhou, China; ^4^Center for Economic Behavior and Decision-Making, Zhejiang University of Finance and Economics, Hangzhou, China; ^5^School of Economics, Zhejiang University of Finance and Economics, Hangzhou, China

**Keywords:** endowment effect, the medial prefrontal cortex, transcranial direct current stimulation, objective value, subjective value

## Abstract

Endowment effect – the observation that people appear to attach more value to possessions than non-possessions – has been replicated in numerous experimental studies. Previous neuroimaging studies revealed that the medial prefrontal cortex (MPFC) plays a role in the endowment effect. To assess the possibility of a direct causal relationship between the activity of MPFC and the endowment effect, we used transcranial direct current stimulation (tDCS) to transiently alter the neural activity in MPFC. Subsequently, in three stimulation treatments, we assessed the presence of the endowment effect, which was demonstrated by a disparity between willingness to accept (WTA) and willingness to pay (WTP). The results indicated that the participants demonstrated the endowment effect for a mug in the anodal and sham treatments, whereas no endowment effect was observed in the cathodal treatment. Similarly, endowment effect was observed for the other item (notebook) in the anodal treatment, whereas no endowment effect was observed in the sham and cathodal treatments. In addition, the participants tended to sell higher and buy lower after receiving anodal tDCS over MPFC and buy higher after receiving cathodal tDCS over MPFC. As a result, the present study demonstrated a direct causal relationship between the activity of MPFC and the endowment effect.

## Introduction

The Coase theorem, one of the best-known theorems in standard economics, states that the allocation of resources will be independent of initial property rights (Coase, 1960). However, numerous behavioral studies have demonstrated that this is not the case. People have a tendency to attach more value to possessions than non-possessions.

This behavioral bias is known as the endowment effect, which was first identified by Richard Thaler. Thaler (1980) found that the minimum compensation participants charged for accepting a 0.001% chance of sudden death was significantly higher than the amount they were willing to pay to eliminate an identical risk. People have a tendency to demand much more to give up an object than to pay for it. There are two classical paradigms (exchange paradigm and valuation paradigm) to show the endowment effect. In the exchange paradigm, half of the participants are randomly endowed with one item, and the other half are randomly endowed with another item. After a few minutes, they can exchange their items for the other item with the experimenter or keep their items. It was reported that the exchange rate among randomly assigned owners was significantly lower than the predicted value (Knetsch and Sinden, 1984). In the valuation paradigm, the participants are randomly assigned to be sellers or buyers, and a significant disparity between the willingness to accept (WTA) and the willingness to pay (WTP) is observed (Kahneman et al., 1990). As opposed to the most fundamental independence assumption in standard economic theory, the endowment effect has attracted enormous attention in behavioral economists since then. It has been widely replicated in numerous settings. In addition to private goods, such as coffee cups, lottery tickets, and chocolates (e.g., Knetsch and Sinden, 1984; Kahneman et al., 1990), people also demonstrate the endowment effect for public goods, such as air quality and guidance services (MacDonald and Bowker, 1994; Bischoff, 2008). Besides adults, young children and non-human primates also demonstrate the same bias (Harbaugh et al., 2001; Venkat et al., 2008). Therefore, it has been called “one of the most important and robust empirical regularities to emerge from the field” and is referred to as “the most robust finding in the psychology of decision making” (Loewenstein and Issacharoff, 1994; Knetsch et al., 2001). However, not all related studies demonstrate the same conclusion. Notably, Plott and Zeiler (2005) identified that this effect might be vulnerable to procedure, whereas Fehr et al. (2015) found no evidence of the idea that subject misconceptions were the main source of the WTA-WTP gap. Given these debates, it is necessary to take a critical attitude toward them.

To distinguish between the different explanations, offer a more convincing account, and clarify the brain representation of the effect, neuroeconomists have investigated it using functional magnetic resonance imaging (fMRI). Previous neuroimaging studies showed that there was a relationship between endowment effect and activity in the neural network, including the nucleus accumbens (NAcc), insula and the medial prefrontal cortex (MPFC) regions (Knutson et al., 2001, 2008; Feng et al., 2013), and subjects showed greater MPFC activation in response to low prices when buying compared with selling (Knutson et al., 2008). Besides, Knutson et al. (2003) reported the role of MPFC in updating the initial predictions of monetary gain. Furthermore, in choice scenarios such as buying, MPFC showed decreased activation in response to excessive prices (Knutson et al., 2007). Neuroimaging studies are useful in establishing correlations, however, these studies did not demonstrate a direct causal relationship between MPFC and the endowment effect. Non-invasive brain stimulation techniques, such as transcranial direct current stimulation (tDCS), can be useful for addressing this question and make it possible to detect its effect on the endowment effect more accurately. Using fMRI, Votinov et al. (2010) found that the brain activation in the right inferior frontal gyrus (IFG) was associated with a price discrepancy; furthermore, the functional relevance of the right IFG in the endowment effect was identified using tDCS (Votinov et al., 2013). However, to the best of our knowledge, tDCS has not been used in assessing a direct causal relationship between MPFC and the endowment effect.

In the current study, we used tDCS to transiently alter the neural activity in the MPFC and then assessed whether there was a significant difference in the disparity between WTA and WTP across three stimulation treatments (anodal tDCS, cathodal tDCS, or sham stimulation). One part of our experiment was based on the valuation paradigm known as the WTA and WTP frame – a valuation task. To assess whether the participants demonstrated the endowment effect for actual ownership or imaginary ownership and clarify the stimulation effects, we chose a mug, which was used in previous studies and designated for actual ownership, and a notebook, which had the same market value as the mug and was designated for imaginary ownership. Besides the valuation task, to assess whether ownership would change their objective value across the three treatments, the participants had to complete an evaluation task to evaluate the same items as those used in the valuation task. Overall, this study aimed to investigate the causal relationship between the MPFC and the endowment effect to explore the microeconomic foundation of the endowment effect from the view of neuroscience.

## Materials and Methods

### Subjects

We recruited 159 healthy college students (107 females; average age 20.43 years old, ranging from 18 to 28 years old) to participate in Experiment 1 and recruited another 60 healthy college students (42 females; average age 21 years old, ranging from 18 to 26 years old) for Experiment 2. All the participants were right-handed and unfamiliar with tDCS, valuation task, and evaluation task, with no history of psychiatric illness or neurological disorders. In Experiment 1, we adopted a 2 × 3 [(selling or buying frame) × (anodal, cathodal, or sham treatment)] between-subjects design. The participants were randomly assigned to receive anodal tDCS and act as sellers (*n* = 28, 19 females), anodal tDCS and act as buyers (*n* = 27, 19 females), cathodal tDCS and act as sellers (*n* = 26, 18 females), cathodal tDCS and act as buyers (*n* = 25, 17 females), sham stimulation and act as sellers (*n* = 27, 18 females), or sham stimulation and act as buyers (*n* = 26, 17 females). In Experiment 2, we adopted a 2 × 2 [(selling or buying frame) × (anodal and sham treatment)] design. The frame was a “between-subjects” design: half of the participants were randomly assigned as buyers, and the other half were assigned as sellers. The stimulation type was a “within-subjects” design: the participants who underwent anodal stimulation for the first time received sham stimulation 1 week later, and the participants who underwent sham stimulation for the first time received anodal stimulation 1 week later. The sequence of two stimulation types was balanced between subjects. The entire experiment lasted about 50 min, and each participant received a payment of approximately 45 CNY (approximately 6.4 US dollars) on average after completing all tasks in one experiment. Participants gave informed written consent before entering the study, which was approved by the Zhejiang University ethics committee. No participants reported any side effects concerning pain on the scalp or headaches after the experiment.

### Transcranial Direct Current Stimulation (tDCS)

Transcranial direct current stimulation (tDCS) applied a weak direct current to the scalp via two saline-soaked surface sponge electrode (35 cm^2^). The current was constant and delivered by a battery-driven stimulator (Starlab, Spain), which was controlled through a Bluetooth signal. Generally speaking, anodal stimulation enhances cortical excitability, whereas cathodal stimulation restrains it (Nitsche and Paulus, 2000).

**FIGURE 1:**
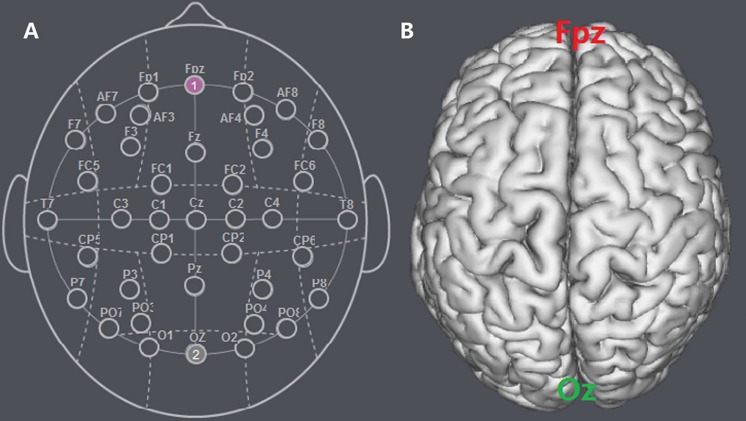
Schematic and locations of the electrode positions. **(A)** Schematic of the electrode positions Fpz and Oz based on the international EEG 10-20 system. **(B)** Locations of the MPFC and the visual cortex (Oz) of the human brain.

**FIGURE 2:**
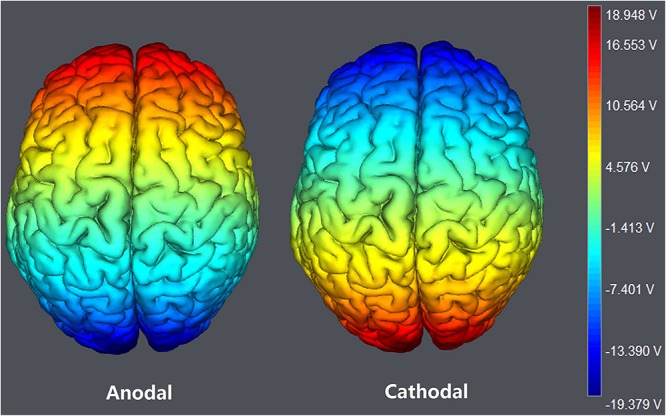
The stimulation models of tDCS treatments. Electric field stimulations were performed with the Neuroelectrics Instrument Controller software (version 1.3, Spain). Stimulated field intensity is indicated by the color bar. The axis represents the range of input voltage from -19.379 to 18.948 V.

Participants were randomly assigned to one of the three stimulation treatments. For anodal stimulation, the anodal electrode was placed over the Fpz according to the international EEG 10-20 system, while the cathodal electrode was placed over the Oz (Figures 1A,B). For cathodal stimulation, the placement was reversed: the anodal electrode was placed over Oz, and the cathodal electrode was placed over Fpz (Figure 2). For sham stimulation, the procedures were the same but the current lasted only for the first 30 s. The participants might have felt the initial itching, but there was actually no current for the rest of the stimulation. This method of sham stimulation has been shown to be reliable (Gandiga et al., 2006). The current was constant and of 1.5 mA intensity with 30 s of ramp up and down, its safety and efficiency were shown in previous studies (Nitsche et al., 2003, 2008).

Before the valuation task, a laboratory assistant put electrodes on a participant’s head for stimulation. After 20 min of stimulation, the participant was asked to complete a valuation task and an evaluation task one by one.

### Task and Procedure

#### Valuation Task

In the valuation task, participants were randomly assigned to one of the two frames. In the WTA frame, the sellers were given a mug that was placed on the seat in front of them, and they were physically given a notebook from the experimenter. In the WTP frame, the buyers were given 20 CNY (approximately 3 US dollars), which equaled to the total market price of one mug and one notebook used in the WTA frame to exclude the income effect.

In this task, based on the classical valuation paradigm with the Becker-Degroot-Marschak (BDM) procedure (Becker et al., 1964) and a multiple-price list, the participants acting as sellers were asked for how little they were willing to sell the mug or the notebook. We asked if they were willing to sell each item at a series of prices ranging from 6 to 15 CNY in increments of 1 CNY to elicit the lowest price they were willing to accept. In the response form, which was written on paper, the participants had to indicate their decision with a tick at each column. A part of the response form for sellers was as follows:

At a price of ¥14, I will sell ____, I will not sell ____;

At a price of ¥15, I will sell ____, I will not sell ____;

I will not sell at any price even if it is larger than 15 ___.

The participants acting as buyers were asked whether they would be willing to buy a mug or a notebook at the same series of prices as those in the WTA frame to elicit the highest price they were willing to pay. In the response form, which was presented on paper, the participants had to indicate their decision with a tick at each column. A part of the response form for buyers was as follows:

I will not buy at any price even if it is less than 6 ____.

At a price of ¥ 6, I will buy ____, I will not buy ____;

At a price of ¥ 7, I will buy ____, I will not buy ____;

#### Evaluation Task

In the evaluation task, they were required to evaluate 5 items in the following sequence: a glove, a desk lamp, a mug, a bicycle lock, and a notebook. A picture of each item appeared on a computer screen one by one and the subjects were required to write their evaluations on paper. The mug and notebook used in this task were the same ones used in the valuation task. Besides the two target items, we selected three more items that are familiar to college students, to avoid making our intentions known to participants. Furthermore, to incentivize the participants to tell their true assessments of these items, they were informed that if the absolute value between their evaluation of each item and its market price were not more than 2 CNY, they could get another 2 CNY for each as a reward.

#### Experimental Procedure

First, the participants were randomly assigned to be sellers or buyers to complete the valuation task. Subsequently, all participants had to complete the evaluation task. After they finished the second task, the participants were asked to complete a questionnaire. The questionnaire contained questions about personal information, such as sex, residence, party membership, student cadre, annual family income, monthly consumption, and monthly online shopping frequency. Furthermore, to make the participants feel like they were in a real-market transaction, at the beginning of the experiment they were informed that they would make real deals with the experimenter; the selling price or buying price from the experimenter was randomly chosen based on the price list used in the valuation task. To incentivize the participants to report their true values, they were informed that if his/her WTA price was not higher than the buying price, he/she could sell the item to the experimenter at the WTA price and not the buying price, and if his/her WTP price was not lower than the selling price, he/she could buy the item from the experimenter at the WTP price and not the selling price. Therefore, in the end, we randomly chose two participants to press the button, showed the selling and buying prices to all the participants, and, finally, calculated their total payments one by one.

As Plott and Zeiler (2005, 2007) identified, the WTA-WTP gap could be due to experimental procedures, such as subject misconceptions stemming from the preference elicitation method, the method and language used to endow subjects, suggestions of relative value, and public revelation of choices. It is necessary to take into account these details when designing methods. In the current study, given the possible influence of subject misconceptions, we adopted the BDM procedure and a multiple price list and added two questions to test whether the participants fully understood the preference elicitation method and the transaction rules. For the sellers, the first question is “if your WTA is 12 CNY and the buying price of the experimenter is 8 CNY, can you sell the item?” The correct answer is no. The other question is “if your WTA is 9 CNY, the buying price should be at least ___ CNY, you can sell the item, and you will get ___ CNY as a part of your payment.” The correct answers are 9 and 9. For the buyers, the first question is “if your WTP is 12 CNY and the selling price of the experimenter is 8 CNY, can you buy the item?” The correct answer is yes. The other question is “if your WTP is 9 CNY, the selling price should be at most ___ CNY, you can buy the item, and you will pay for ___ CNY.” The correct answers are 9 and 9. Only by answering these questions correctly (meaning they fully understood the main procedure), could the participants enter the formal valuation task. Then, participants’ roles were randomly selected by themselves rather than by the experimenter, and the language used to endow items was neutral. Moreover, all participants reported their choices privately.

**FIGURE 3:**
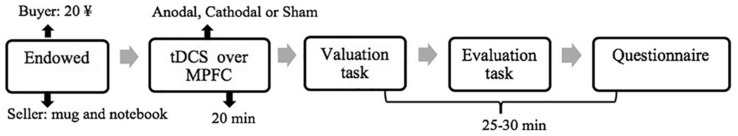
Schematic representation of the experimental design in Experiment 1. After 20 min of stimulation, the participant was asked to complete a valuation task and an evaluation task one by one.

**FIGURE 4:**
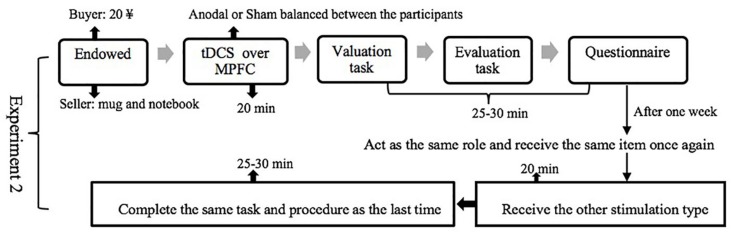
Schematic representation of the experimental design in Experiment 2. The participants had to come to the lab twice and complete the same task and procedure as that in the Experiment 1 after receiving one of two stimulation types (anodal or sham).

In Experiment 1, after receiving one of three stimulation types (anodal, cathodal, or sham) for 20 min, the participants were asked to complete the valuation task and the evaluation task one by one (Figure 3). In Experiment 2, the participants had to come to the lab twice and complete the same task and procedure as that in Experiment 1 after receiving one of two stimulation types (anodal or sham). The participants who underwent anodal stimulation in the first session were assigned to receive sham stimulation in the second session, and the participants who underwent sham stimulation in the first session were assigned to receive anodal stimulation in the second session. In order to decrease the learning effect, the sequence of two stimulation types was balanced between subjects and the interval between two sessions was 1 week (Figure 4).

### Data Analysis

In Experiment 1, we ran an ANOVA with the frame (WTA and WTP frame) and tDCS stimulation type (anodal, cathodal and sham) as “between-subjects” factors, and the offers (WTA and WTP) for the mug (the offers for the notebook, the evaluations of the mug, or the evaluations of the notebook) as dependent variables. If both the main and interaction effects were significant, then we compared WTA and WTP in different stimulation groups to explore the effect of tDCS in the MPFC on the endowment effect, and tested whether WTA or WTP was significantly different among the three stimulation types (anodal, cathodal, and sham) via *post hoc* analyses (Bonferroni) within ANOVA to clarify the stimulation effect. If the main effects were significant, we made comparisons at the level of the frame and stimulation type separately via ANOVA. Otherwise, we concluded that there was no significant difference in each comparison.

In Experiment 2, we first ran repeated ANOVA with the frame (WTA and WTP frame) as a “between-subjects” factor and tDCS stimulation type (anodal and sham) as a “within-subjects” factor to test the interaction effect in each task. If the interaction effect was significant, then we conducted a one-way ANOVA to test the effect of the frame in each stimulation type, and we also tested the effect of the stimulation in each frame with a paired *T*-test.

All data were statistically evaluated using SPSS software (version 23, SPSS Inc., Chicago, IL, United States). The significance level was set at 0.05 for all analyses. Means and standard errors of the WTA, WTP, evaluation in the WTA frame, and evaluation in the WTP frame are shown in Table 1 (mug) and Table 2 (notebook).

**TABLE 1:** Means and standard errors of the data for the mug in Experiment 1.

**Stimulation**	**Anodal**		**Sham**		**Cathodal**	
**Mug**	***M***	***SE***	***M***	***SE***	***M***	***SE***
WTA	10.39	0.27	9.44	0.30	9.42	0.36
WTP	7.56	0.42	7.35	0.36	9.52	0.56
Eva in WTA frame	13.18	0.77	12.37	0.86	13.65	1.22
Eva in WTP frame	12.74	0.81	12.15	0.76	13.40	0.87

**TABLE 2:** Means and standard errors of the data for the notebook in Experiment 1.

**Stimulation**	**Anodal**		**Sham**		**Cathodal**	
**Notebook**	***M***	***SE***	***M***	***SE***	***M***	***SE***
WTA	10.79	0.40	10.59	0.45	11.69	0.55
WTP	8.56	0.52	10.08	0.50	10.72	0.61
Eva in WTA frame	15.71	1.16	15.26	1.05	16.96	1.47
Eva in WTP frame	13.30	0.76	15.15	0.99	15.64	1.20

## Results

### Experiment 1

#### Valuation Task

**FIGURE 5:**
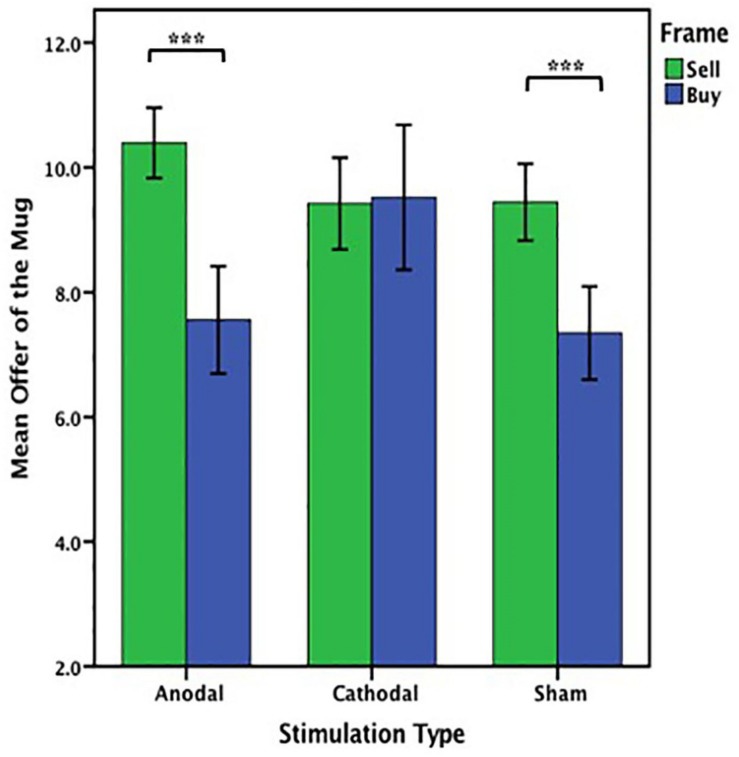
Data of offer of the mug (Experiment 1). The mean offer of the mug across three stimulations in each frame. Error bars indicate 95% confidence intervals. Asterisks indicate statistically significant difference between WTA and WTP.

**FIGURE 6:**
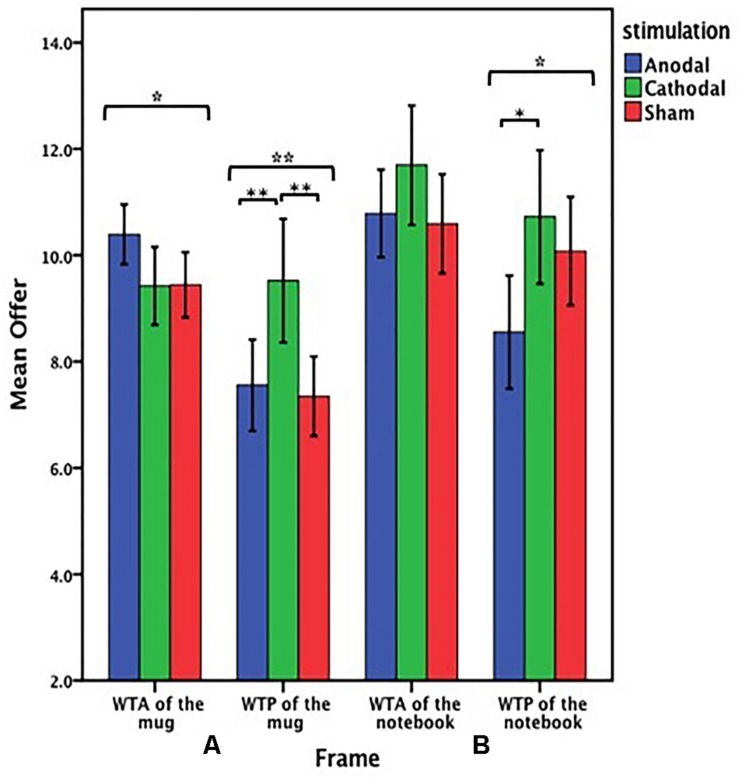
Data of offer in the valuation task (Experiment 1). **(A)** The mean offer of the mug across three stimulations in each frame. **(B)** The mean offer of the notebook across three stimulations in each frame. Error bars indicate 95% confidence intervals. Asterisks indicate statistically significant difference between the treatments.

The offers (WTA and WTP) on the mug from participants receiving anodal and cathodal tDCS over MPFC and the sham groups were analyzed by ANOVA with the frame (WTA and WTP frame) and tDCS stimulation type (anodal, cathodal and sham) as “between-subjects” factors. A significant main effect of frame [*F*_(1, 153)_ = 26.313, *p* < 0.001, partial η^2^ = 0.147] and a significant main effect of stimulation type [*F*_(__2__,153__)_ = 3.846, *p* = 0.023, partial η^2^ = 0.048] were observed. Notably, there was also a significant interaction effect involving the frame and stimulation type [*F*_(__2__,153__)_ = 7.763, *p* = 0.001, partial η^2^ = 0.092]. A significant difference between WTA and WTP was found in the anodal group [*F*_(__1__,53__)_ = 32.701, *P* < 0.001] and the sham group [*F*_(__1__,51__)_ = 20.021, *P* < 0.001], but there was no significant difference between WTA and WTP in the cathodal group [*F*_(__1__,49__)_ = 0.022, *P* = 0.884] (Figure 5). We found that both WTA [*F*_(__2__,78__)_ = 3.259, *p* = 0.044] and WTP [*F*_(__2__,75__)_ = 6.881, *p* = 0.002] were significantly different among the three groups (Figure 6A). *Post hoc* analyses (Bonferroni) revealed that in the WTA frame, WTA of the mug obtained in the anodal group (mean = 10.393) were slightly higher than that obtained in the cathodal group (mean = 9.423, *p* = 0.089) or the sham group (mean = 9.444, *p* = 0.095). There was no significant difference between the cathodal group and the sham group (*p* = 1.000). Moreover, in the WTP frame, WTP of the mug obtained in the cathodal group (mean = 9.520) were significantly higher than that obtained in the anodal group (mean = 7.556, *p* = 0.009) or the sham group (mean = 7.346, *p* = 0.004). No significant difference between the anodal group and the sham group was observed (*p* = 1.000) (Figure 6A).

To assess whether the participants demonstrated the endowment effect for imaginary ownership and to clarify the stimulation effect, the offers (WTA and WTP) on the notebook from all the stimulation groups were analyzed by ANOVA with the frame (WTA and WTP frame) and tDCS stimulation type (anodal, cathodal, and sham) as “between-subjects” factors. There was no significant interaction effect involving the frame and stimulation type [*F*_(2,153)_ = 1.581, *p* = 0.209, partial η^2^ = 0.020], but a significant main effect of frame [*F*_(1,153)_ = 9.038, *p* = 0.003, partial η^2^ = 0.056] and a significant main effect of stimulation type [*F*_(2,153)_ = 4.637, *p* = 0.011, partial η^2^ = 0.057] were observed.

**FIGURE 7:**
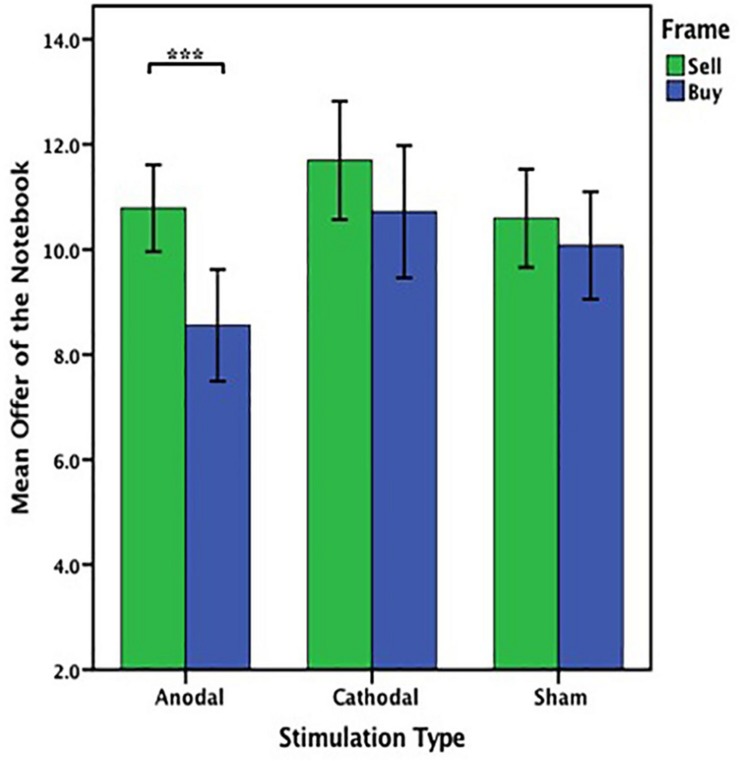
Data of offer of the notebook (Experiment 1). The mean offer of the notebook across three stimulations in each frame. Error bars indicate 95% confidence intervals. Asterisks indicate statistically significant difference between WTA and WTP.

Based on the main effect by stimulation type on the offers on the notebook, the offers on the notebook from the anodal group were analyzed by ANOVA with the frame (WTA and WTP frame) as a “between-subjects” factor. There was a significant influence of the frame in the anodal stimulation [*F*_(1,53)_ = 11.636, *P* = 0.001, partial η^2^ = 0.180]. The offers of the notebook from the cathodal group or the sham group were analyzed as before; no significant effect of the frame was observed either in the cathodal group [*F*_(1,49)_ = 1.417, *P* = 0.240, partial η^2^ = 0.028] or the sham group [*F*_(1,51)_ = 0.590, *P* = 0.446, partial η^2^ = 0.011] (Figure 7).

Based on the main effect of the frame on the offers on the notebook, WTA of the notebook was analyzed by ANOVA with the tDCS stimulation type (anodal, cathodal and sham) as a “between-subjects” factor. There was no significant influence of stimulation type [*F*_(2,78)_ = 1.542, *p* = 0.220, partial η^2^ = 0.038]. WTP of the notebook was also analyzed by ANOVA with the tDCS stimulation type (anodal, cathodal, and sham) as a “between-subjects” factor. A significant influence of stimulation type was observed [*F*_(2,75)_ = 4.253, *p* = 0.018, partial η^2^ = 0.102]. *Post hoc* analyses (Bonferroni) showed that in the WTP frame, WTP of the notebook obtained in the anodal group (mean = 8.556) was significantly lower than that obtained in the cathodal group (mean = 10.720, *p* = 0.018). However, there were no significant differences between the cathodal group (mean = 10.720) and the sham group (mean = 10.077, *p* = 1.000) or the anodal group (mean = 8.556) and the sham group (mean = 10.077, *p* = 0.145) (Figure 6B).

#### Evaluation Task

The evaluations of the mug and notebook were analyzed by ANOVA with the frame (WTA and WTP frame) and tDCS stimulation type (anodal, cathodal and sham) as “between-subjects” factors. Neither a main effect of frame [Mug: *F*_(1,153)_ = 0.173, *p* = 0.678, partial η^2^ = 0.001; Notebook: *F*_(1,153)_ = 1.956, *p* = 0.164, partial η^2^ = 0.013] or stimulation type [Mug: *F*_(2,153)_ = 0.994, *p* = 0.372, partial η^2^ = 0.013; Notebook: *F*_(2,153)_ = 1.293, *p* = 0.277, partial η^2^ = 0.017] nor a significant interaction effect involving the frame and stimulation type [Mug: *F*_(2,153)_ = 0.009, *p* = 0.991, partial η^2^ < 0.001; Notebook: *F*_(2,153)_ = 0.541, *P* = 0.583, partial η^2^ = 0.007] was observed. The results of the evaluations of the other three items (glove, desk lamp, and bicycle lock) which were selected to avoid making our intentions known to the participants are presented in Supplementary Material.

We also tested for a possible effect of demographic characteristics (sex, residence, party membership, student cadre, annual family income, monthly consumption, and monthly online shopping frequency) on the dependent variables (offers and evaluations) when entered in the model as covariates. Apart from a significant effect of sex on the offers on the mug [*F*_(1,146)_ = 9.522, *p* = 0.002, partial η^2^ = 0.061] and a significant effect of sex on the evaluation of the notebook [*F*_(1,146)_ = 11.865, *p* = 0.001, partial η^2^ = 0.075], no significant effect was observed.

We found that participants who underwent the sham stimulation demonstrated the endowment effect of the mug, but not for the notebook. The participants who underwent the anodal stimulation demonstrated the endowment effect for both the mug and notebook, whereas the participants in the cathodal group did not for neither the mug nor notebook. Notably, for the mug, the average of the WTP (mean = 9.520) was numerically larger than the average of the WTA (mean = 9.423) in the cathodal group. Furthermore, the participants acting as sellers tended to sell higher for the mug after receiving anodal stimulation and the participants acting as buyers tended to buy lower for both the mug and notebook after receiving anodal stimulation. The participants tended to buy higher for both items after receiving cathodal stimulation. However, there was no significant difference in the evaluation between the two frames across the three stimulation groups in the evaluation task.

### Experiment 2

#### Valuation Task

**FIGURE 8:**
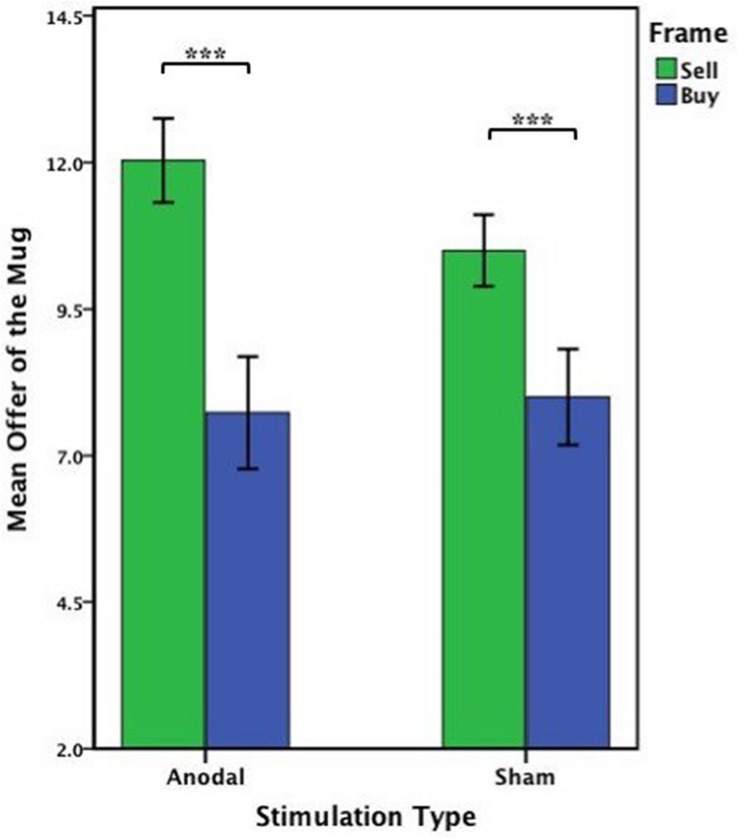
Data of offer of the mug (Experiment 2). The mean offer of the mug across anodal or sham stimulation in each frame. Error bars indicate 95% confidence intervals. Asterisks indicate statistically significant difference between WTA and WTP.

**FIGURE 9:**
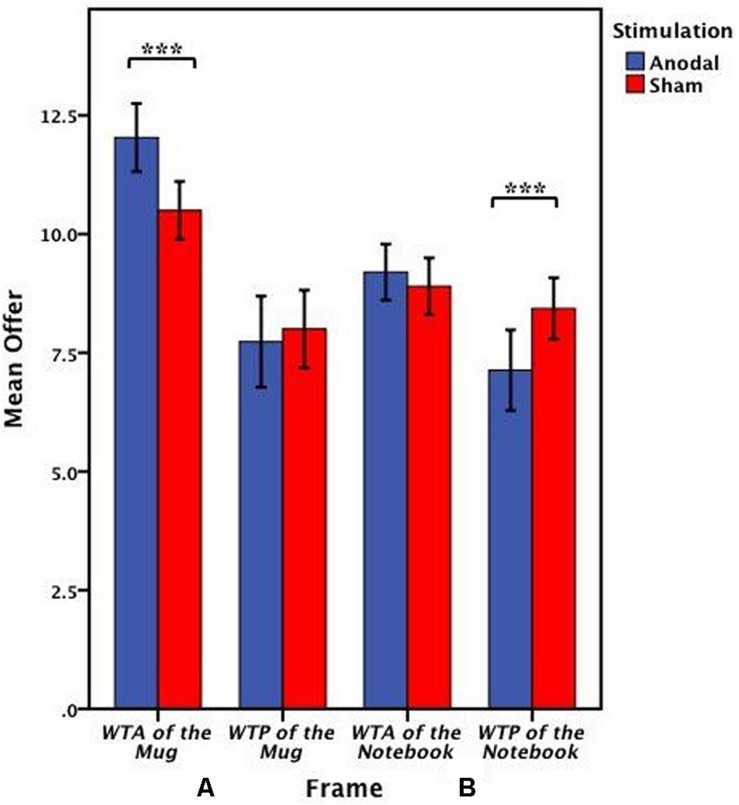
Data of offer in the valuation task (Experiment 2). **(A)** The mean offer of the mug across anodal or sham stimulation in each frame. **(B)** The mean offer of the notebook across anodal or sham stimulation in each frame. Error bars indicate 95% confidence intervals. Asterisks indicate statistically significant difference between the treatments.

The offers (WTA and WTP) on the mug from the participants receiving anodal tDCS over MPFC and sham groups were analyzed by repeated ANOVA with the frame (WTA and WTP frame) as a “between-subjects” factor and tDCS stimulation type (anodal and sham) as a “within-subjects” factors. There were significant main effects of the frame [*F*_(1,58)_ = 52.701, *p* < 0.001, partial η^2^ = 0.476] and tDCS stimulation type [*F*_(1,58)_ = 5.183, *p* = 0.027, partial η^2^ = 0.082]. Notably, a significant interaction effect involving the frame and stimulation type was observed [*F*_(1,58)_ = 10.446, *p* = 0.002, partial η^2^ = 0.153]. A significant difference between WTA and WTP was found both in the anodal group [one-way ANOVA, *F*_(1,58)_ = 53.830, *p* < 0.001] and the sham group [one-way ANOVA, *F*_(1,58)_ = 25.000, *p* < 0.001] (Figure 8). The participants acting as sellers tended to sell higher for the mug after receiving anodal stimulation (mean = 12.033) than those receiving sham stimulation (mean = 10.500) (paired *T*-test, *t*_29_ = 4.490, *p* < 0.001), whereas there was no significant difference in WTP between anodal (mean = 7.733) and sham stimulations (mean = 8.000) (paired *T*-test, *t*_29_ = -0.607, *p* = 0.549) (Figure 9A).

**FIGURE 10:**
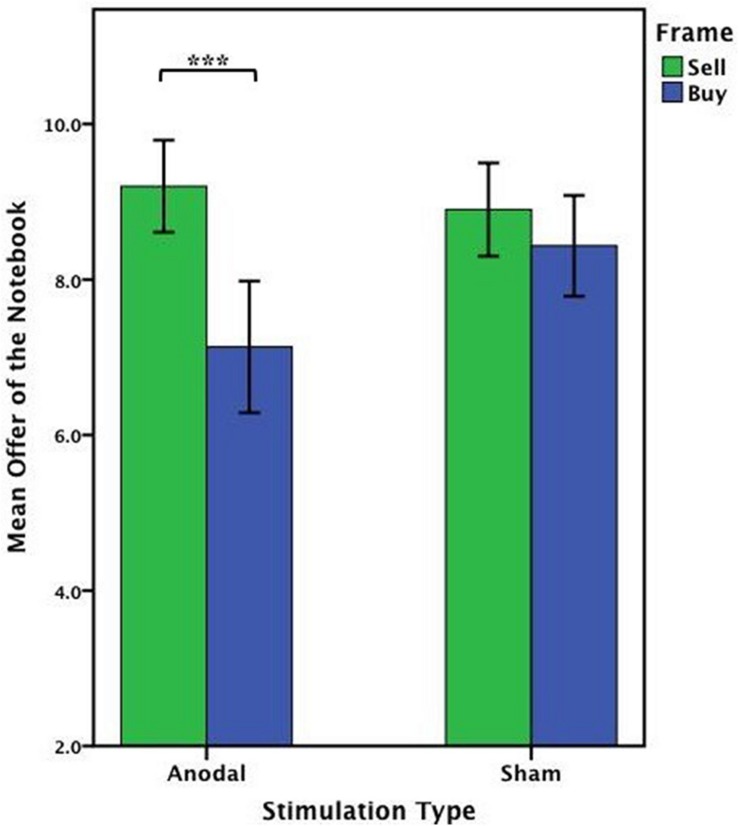
Data of offer of the notebook (Experiment 2). The mean offer of the notebook across anodal or sham stimulation in each frame. Error bars indicate 95% confidence intervals. Asterisks indicate statistically significant difference between WTA and WTP.

The offers (WTA and WTP) on the notebook from the participants receiving anodal tDCS to the MPFC and sham groups were also analyzed by repeated ANOVA with the frame (WTA and WTP frame) as a “between-subjects” factor and tDCS stimulation type (anodal and sham) as a “within-subjects” factors. There were significant main effects of the frame [*F*_(1,58)_ = 8.162, *p* = 0.006, partial η^2^ = 0.123] and tDCS stimulation type [*F*_(1,58)_ = 10.284, *p* = 0.002, partial η^2^ = 0.151]. Notably, a significant interaction effect involving the frame and stimulation type was also observed [*F*_(1,58)_ = 26.326, *p* < 0.001, partial η^2^ = 0.312]. A significant difference between WTA and WTP was found in the anodal group [one-way ANOVA, *F*_(1,58)_ = 16.718, *p* < 0.001], but there was no significantly difference between WTA and WTP in the sham group [one-way ANOVA, *F*_(1,58)_ = 1.169, *p* = 0.284] (Figure 10). The participants acting as buyers tended to buy lower for the notebook after receiving anodal stimulation (mean = 7.133) than those receiving sham stimulation (mean = 8.433) (paired *T*-test, *t*_29_ = -5.204, *p* < 0.001), whereas there was no significant difference in WTA between anodal (mean = 9.20) and sham stimulations (mean = 8.90) (paired *T*-test, *t*_29_ = 1.608, *p* = 0.119) (Figure 9B).

#### Evaluation Task

The evaluations of the mug and notebook were analyzed via repeated ANOVA with the frame (WTA and WTP frame) as a “between-subjects” factor and tDCS stimulation type (anodal, cathodal, and sham) as a “within-subjects” factor. Neither a main effect of frame [Mug: *F*_(1,58)_ = 0.926, *p* = 0.340, partial η^2^ = 0.016; Notebook: *F*_(1,58)_ = 0.022, *p* = 0.883, partial η^2^ < 0.001] or stimulation type [Mug: *F*_(1,58)_ = 1.596, *p* = 0.212, partial η^2^ = 0.027; Notebook: *F*_(1,58)_ = 0.242, *p* = 0.625, partial η^2^ = 0.004] nor a significant interaction effect involving the frame and stimulation type [Mug: *F*_(1,58)_ = 0.623, *p* = 0.433, partial η^2^ = 0.011; Notebook: *F*_(1,58)_ = 0.061, *p* = 0.807, partial η^2^ = 0.001] was observed. The results of the evaluations of the other three items (glove, desk lamp, and bicycle lock) which were selected to avoid making our intentions known to the participants are presented in Supplementary Material.

We found that participants demonstrated the endowment effect on the mug, but not for the notebook, after receiving sham stimulation. After receiving anodal stimulation, they demonstrated the effect for both the mug and notebook. Furthermore, the participants tended to sell higher for the mug after receiving anodal stimulation than receiving sham stimulation, and they tended to buy lower for the notebook after receiving anodal stimulation than receiving sham stimulation. However, there was no significant difference in the evaluation between the two frames across the three stimulation groups in the evaluation task. These results shown in Experiment 2 were consistent with the findings in Experiment 1.

## Discussion

### Valuation Task

In the present study, to assess whether the participants demonstrated the endowment effect for actual ownership or imaginary ownership, we adopted two items that had the same market value and were received from the same experimenter, however, one was placed in the seat before a participant and the other was kept by the experimenter during the experiment. We found that participants who underwent sham stimulation demonstrated the endowment effect for the mug, but not for the notebook, which was different from the findings of some previous studies (Furby, 1980; Pierce et al., 2003).

Based on these findings in the sham group, we assessed the stimulation effect. For the mug, participants in the anodal group demonstrated the endowment effect, whereas they had no endowment effect for the mug after receiving cathodal tDCS over MPFC. In addition to these changes in the endowment effect, the average WTA (mean = 9.423) of the mug was less than the average WTP (mean = 9.520) in the cathodal group. As for the notebook, the participants demonstrated the endowment effect after receiving anodal tDCS over MPFC, whereas the participants who underwent cathodal stimulation did not, which was consistent with the tendencies observed for the mug. Compared with the findings in the sham group, we concluded that restraining the activity in the MPFC resulted in the disappearance of the endowment effect for both items, while stimulating the activity in the MPFC resulted in the endowment effect for the notebook, which was designed as imaginary ownership.

As shown in previous neuroimaging studies, increased activation in the MPFC is found more during self-related judgments than during other-related judgments (Craik et al., 1999; Fossati et al., 2004), the MPFC is engaged in representation of self-knowledge (Zhu et al., 2007), and the MPFC is necessary for the self-reference effect (SRE) and is important for self-referential processing and the neural representation of self (Philippi et al., 2012). Consequently, when it comes to possessions, people might regard them as representations of themselves, and demonstrate the endowment effect for their possessions. As for the stimulation effect, after cathodal stimulation, the activation of the MPFC was decreased. The feeling of self-knowledge might reduce, yielding no significant WTA-WTP gap in the cathodal group. After anodal stimulation, the activation of the MPFC was enhanced. The feeling of self-knowledge might increase, and then subjects might demonstrate the endowment effect for imaginary ownership, while there was no endowment effect for imaginary ownership in the sham stimulation group.

Furthermore, because of the valuation paradigm we adopted in the valuation task, we had the opportunity to detect active stimulation effects in WTA and WTP frames, and explore the causes of changes in the endowment effect. Our findings indicated that for the mug, the participants who received cathodal tDCS over MPFC sold lower and bought higher than those who received anodal tDCS over MPFC. They also bought higher than those in the sham group, which might explain the disappearance of the endowment effect for the mug in the cathodal group. Additionally, the participants who received anodal tDCS over MPFC also sold higher than those in the sham group. As for the notebook, the participants who received anodal tDCS over MPFC bought lower than the others in the cathodal or sham groups, which might have resulted in the endowment effect for the notebook in the anodal group, whereas there was no endowment effect in the cathodal and sham groups. Overall, the participants tended to sell higher and buy lower after receiving anodal tDCS over MPFC, and they were willing to sell lower and buy higher after cathodal tDCS over MPFC. In the current study, sellers in the anodal stimulation group were more reluctant to sell than the other two groups, which was consistent with the finding that sellers acted more differently from choosers than buyers did (Kahneman et al., 1990). Moreover, we found that buyers in the cathodal stimulation group were more willing to buy than the other two groups.

Neuroimaging studies have also indicated a correlation between MPFC activation and the endowment effect. MPFC activation has been implicated in updating initial predictions of monetary gain (Knutson et al., 2003). In choice scenarios such as buying, the MPFC showed decreased activation in response to excessive price (Knutson et al., 2007). Additionally, people showed decreased MPFC activation to low prices when selling, but showed increased MPFC activation to low prices when buying. In other words, people showed greater MPFC activation in response to low prices when buying compared with selling (Knutson et al., 2008). Based on these findings, we hypothesized that in choice scenarios such as selling, after enhancing MPFC activation, subjects might sell items at higher prices, and in choice scenarios such as buying, after restraining MPFC activation, subjects might buy items at higher prices, which were consistent with the results of our findings.

To conclude, not only did these findings support the conclusions of some neuroimaging studies that MPFC plays a crucial role in the endowment effect, but also demonstrated a direct causal relationship between MPFC and the endowment effect.

### Evaluation Task

In contrast to the significant differences in the frame and stimulation types observed in the valuation task, there was no significant difference in the evaluation between the two frames across the three stimulation treatments in the evaluation task in regards to the mug and the notebook. These results indicated that once owned, the participants attached more to subjective value but not objective value, and stimulating or restraining the activity in the MPFC would only play a role in the participant’s subjective value. Consequently, the ownership and change in the activity of the MPFC could not change the objective value; therefore, the disparity between WTA and WTP did not result from a change in the evaluations, rather only ownership made a difference in the endowment effect. Furthermore, one previous study indicated that product ownership of a good had a positive effect on people’s economic valuation of the good, while it had no significant effect on people’s attitudes (De Groot et al., 2009). Our finding in the evaluation task was consistent with the finding that ownership did not affect attitude toward the good, but made a difference in the endowment effect.

### Limitations

The limitation of the present study was that all tasks were performed on paper; therefore, we could not collect the participants’ reaction times, and compare them between the sellers and buyers to assess whether ownership will make them think twice before they made a decision, which might be another method to demonstrate the endowment effect. Therefore, future study may use the computer program for the task. Furthermore, future studies may include other non-invasive brain stimulation techniques, such as transcranial alternating current stimulation (tACS) (see Herrmann et al., 2013, for a review), and neuroimaging measures to explore the neural changes that are associated with neuromodulation leading to behavioral effects.

## Conclusion

In this study, for the mug that represented actual ownership, the participants demonstrated the endowment effect in the anodal and sham treatments, whereas the participants in the cathodal treatment did not. For the notebook that represented imaginary ownership, the endowment effect was observed in the anodal treatment, whereas no endowment effect was observed in the sham and cathodal treatments. Additionally, the participants tended to sell higher and buy lower after receiving anodal tDCS over MPFC, and buy higher after cathodal tDCS over MPFC, which might explain the changes in the endowment effect between the different treatments. Furthermore, there was no significant difference in subjects’ objective values between the two frames across the three treatments. In other words, ownership could make a difference in their subjective value, but had no influence on the objective value. In conclusion, our findings demonstrated a direct causal relationship between the activity of the MPFC and the endowment effect and provided a microeconomic foundation of the endowment effect from the view of neuroscience, which might explain its evolutionary significance. Apicella et al. (2014) demonstrated evolutionary origins of the endowment effect based on evidence from hunter-gatherers. Evolutionary origins have been an explanation of this effect (Harbaugh et al., 2001; Venkat et al., 2008). In the current study, based on the correction between the MPFC and this effect, we demonstrated a causal relationship between them, which might offer evidence for its evolutionary significance.

## Data Availability

All datasets generated for this study are included in the manuscript/Supplementary Files.

## Ethics Statement

This study was carried out in accordance with the recommendations of the guideline of tDCS experiment, Zhejiang University ethics committee with written informed consent from all subjects. All subjects gave written informed consent in accordance with the Declaration of Helsinki. The protocol was approved by the Zhejiang University ethics committee.

## Author Contributions

WG, JS, XL, HY, and JL designed the experiment and wrote the manuscript. WG, JS, YH, and JL performed the experiment. WG, JS, and JL analyzed the data. WG, HY, and JL draw the figures.

## Conflict of Interest Statement

The authors declare that the research was conducted in the absence of any commercial or financial relationships that could be construed as a potential conflict of interest.
