# Supplementary material for: Modulating the Activity of MPFC With tDCS Alters Endowment Effect
Source: Front Behav Neurosci. 2019 Sep 12;13:211. doi: 10.3389/fnbeh.2019.00211 (PMC6751267; doi:10.3389/fnbeh.2019.00211)
Supplement: Supplementary file 1 [file Data_Sheet_1.docx]

# Supplementary Material

**The results of the other three items:**

**Experiment 1**

**Evaluation task**

As for the other three items (glove, desk lamp and bicycle lock), the evaluation of them were also analyzed by ANOVA with the frame (WTA and WTP frame) and tDCS stimulation type (anodal, cathodal and sham) as “between-subjects” factors. Neither a main effect of frame [Glove: F _(1, 153)_ = 0.018, p = 0.895, partial η^2^ < 0.001; Lamp: F _(1, 153)_ = 0.037, p = 0.848, partial η^2^ < 0.001; Lock: F _(1, 153)_ = 0.735, p = 0.393, partial η^2^ = 0.005] or stimulation type [Glove: F _(1, 153)_ = 1.677, p = 0.190, partial η^2^ ＝ 0.021; Lamp: F _(1, 153)_ = 0.407, p = 0.667, partial η^2^ ＝ 0.005; Lock: F _(1, 153)_ = 0.569, p = 0.567, partial η^2^ ＝ 0.007] nor a significant interaction effect involving the frame and stimulation type [Glove: F _(1, 153)_ = 1.465, p = 0.234, partial η^2^ ＝ 0.019; Lamp: F _(1, 153)_ = 1.708, p = 0.185, partial η^2^ ＝ 0.022; Lock: F _(1, 153)_ = 1.040, p = 0.356, partial η^2^ ＝ 0.013] was observed.

**Experiment 2**

**Evaluation task**

We also tested the evaluation of the other three items (glove, desk lamp and bicycle lock) by repeated ANOVAs with the frame (WTA and WTP frame) as a “between-subjects” factor and tDCS stimulation type (anodal, cathodal and sham) as a “within-subjects” factor. Neither a main effect of frame [Glove: F _(1, 58)_ = 0.672, p = 0.416, partial η^2^ ＝ 0.011; Lamp: F _(1, 58)_ = 0.771, p = 0.384, partial η^2^ ＝ 0.013; Lock: F _(1, 58)_ = 0.981, p = 0.326, partial η^2^ ＝ 0.017] or stimulation type [Glove: F _(1, 58)_ = 0.032, p = 0.858, partial η^2^ ＝ 0.001; Lamp: F _(1, 58)_ = 0.624, p = 0.433, partial η^2^ ＝ 0.011; Lock: F _(1, 58)_ = 0.031, p = 0.861, partial η^2^ ＝ 0.001] nor a significant interaction effect involving the frame and stimulation type [Glove: F _(1, 58)_ = 2.460, p = 0.122, partial η^2^ ＝ 0.041; Lamp: F _(1, 58)_ = 1.671, p = 0.201, partial η^2^ ＝ 0.028; Lock: F _(1, 58)_ = 2.354, p = 0.130, partial η^2^ ＝ 0.039] was observed.
